# Overexpression of Nfe2l1 increases proteasome activity and delays vision loss in a preclinical model of human blindness

**DOI:** 10.1126/sciadv.add5479

**Published:** 2023-07-14

**Authors:** Yixiao Wang, Aaron Snell, Frank M. Dyka, Elizabeth R. Colvin, Cristhian Ildefonso, John D. Ash, Ekaterina S. Lobanova

**Affiliations:** ^1^ Department of Ophthalmology, University of Florida, Gainesville, FL 32610, USA.; ^2^ Department of Ophthalmology, University of Pittsburgh, Pittsburgh, PA 15260, USA.; ^3^ Department of Pharmacology and Therapeutics, University of Florida, Gainesville, FL 32610, USA.

## Abstract

Proteasomes are the central proteolytic machines that are critical for breaking down most of the damaged and abnormal proteins in human cells. Although universally applicable drugs are not yet available, the stimulation of proteasomal activity is being analyzed as a proof-of-principle strategy to increase cellular resistance to a broad range of proteotoxic stressors. These approaches have included the stimulation of proteasomes through the overexpression of individual proteasome subunits, phosphorylation, or conformational changes induced by small molecules or peptides. In contrast to these approaches, we evaluated a transcription-driven increase in the total proteasome pool to enhance the proteolytic capacity of degenerating retinal neurons. We show that overexpression of nuclear factor erythroid-2-like 1 (Nfe2l1) transcription factor stimulated proteasome biogenesis and activity, improved the clearance of the ubiquitin-proteasomal reporter, and delayed photoreceptor neuron loss in a preclinical mouse model of human blindness caused by misfolded proteins. The findings highlight Nfe2l1 as an emerging therapeutic target to treat neurodegenerative diseases linked to protein misfolding.

## Teaser

Increasing amounts of proteasomes might help to mitigate human diseases linked to proteostasis dysregulation.

## INTRODUCTION

Alterations in the ability of cells to maintain a healthy proteome are thought to contribute to the pathogenesis of multiple human diseases and aging (*1*–*3*). Most of the proteins in human cells are degraded by the ubiquitin-proteasome system (UPS), with proteasomes being essential proteolytic machines cleaving proteins into smaller polypeptides (*4*). A proteasome consists of several principal components. The core 20*S* particle of a proteasome contains proteolytic sites, which become accessible after association with the 19*S* cap, facilitating the degradation of polyubiquitinated proteins or 11S and PA200 regulators, allowing the degradation of polypeptides and unstructured proteins not modified with ubiquitin (*5*–*9*). An increasing number of studies have demonstrated that stimulation of proteasomes increases cell resistance to various types of proteotoxic stressors and delays aging (*10*–*16*). Some of the investigated approaches to stimulating proteasomes include overexpression of individual proteasome subunits (e.g., PSMD11, β5, 11Sα, and α3ΔΝ), modulation of proteasome activity through phosphorylation, and the development of small compounds and peptides capable of opening 20*S* particles to allow access to their proteolytic sites by certain protein substrates (*4*, *15*–*21*).

An alternative approach to increase the proteolytic capacity of cells and treat human diseases caused by protein misfolding might include increasing the total proteasome pool. Several studies in diverse experimental systems, including mouse fibroblasts, the brain, the retina, and muscles, reported higher levels of proteasomes under genetic activation of the mTORC1 (mechanistic target of rapamycin complex 1) pathway (*22*–*26*). The mechanisms driving this transcriptional program have not been fully characterized but are proposed to be triggered by sterol-regulatory element binding protein 1 (Srebp1)–mediated transcriptional up-regulation of the nuclear factor erythroid-2-like 1 (Nfe2l1) transcription factor (*22*, *27*). Drugs that increase proteasome activity through Nfe2l1-mediated proteasomal transcription have not yet been developed. Building on these findings, we previously examined the genetic activation of the mTORC1 pathway to increase proteasome levels and treat retinal diseases (*24*). Although we observed elevated levels of Nfe2l1 transcripts and proteasomes in rod photoreceptor–specific Tsc2 (tuberous sclerosis complex 2)–knockout mice (Tsc2^Rod KO^), this transcriptional response was suppressed in degenerating rods stressed by misfolded proteins (*24*). Recent studies indicated that overexpression of Nfe2l1 can raise proteasomal levels in vivo in brown fat and cardiomyocytes in mice (*28*, *29*). Therefore, here, we examined direct Nfe2l1 overexpression as an alternative approach to increase the pool of proteasomes in neurodegenerative diseases linked to impaired proteostasis in the retina.

We report the critical role of Nfe2l1 in the control of proteasomal levels in the retina: Its overexpression increases, and knockout reduces the proteasomal pool and activity. We found that overexpression of Nfe2l1 is not toxic to the retina and improved clearance of in vivo UPS reporter in photoreceptors of mice struggling with misfolded proteins, supporting an augmentation of Nfe2l1 pathway as a potent approach to stimulate degradation of ubiquitinated proteins. Finally, we showed that Nfe2l1 overexpression delayed visual loss in a preclinical model of human blindness caused by a misfolded protein called rhodopsin. The findings position the Nfe2l1 pathway as an emerging target for drug development and focus on enhancing this pathway to treat diseases caused by dysregulated proteostasis with potential applications extending beyond retinal pathologies.

## RESULTS

### Nfe2l1 sets proteasomal levels and activity in the retina

To identify the role of Nfe2l1 in control of proteasomal levels in the retina, we took advantage of a previously developed transgenic mouse (hereafter Nfe2l1^OE^ mice) driving expression of *Nfe2l1* under control of the broadly active promoter of *MafG* gene (*musculo-aponeurotic fibrosarcoma gene*) (*30*). These mice, which were developed to study glucose metabolism in the liver, were viable and fertile and rescued otherwise lethal whole-body knockout of the *Nfe2l1* gene (*30*). In the following experiments, we examined changes in proteasomal levels in the retinas and livers of Nfe2l1^OE^ mice. We used retinal lysates prepared from mice lacking *Nfe2l1* gene in retina to control for the specificity of anti-Nfe2l1 antibodies and to determine the contribution of basal Nfe2l1 activity to defining proteasome levels. Retina-specific Nfe2l1-knockout mice (hereafter, Nfe2l1^Retina KO^ mice) were generated by crossing mice bearing the floxed *Nfe2l1* allele and *Chx10-Cre* mice expressing Cre recombinase in all retinal neurons and Müller cells early in development (*31*).

**Fig. 1.**
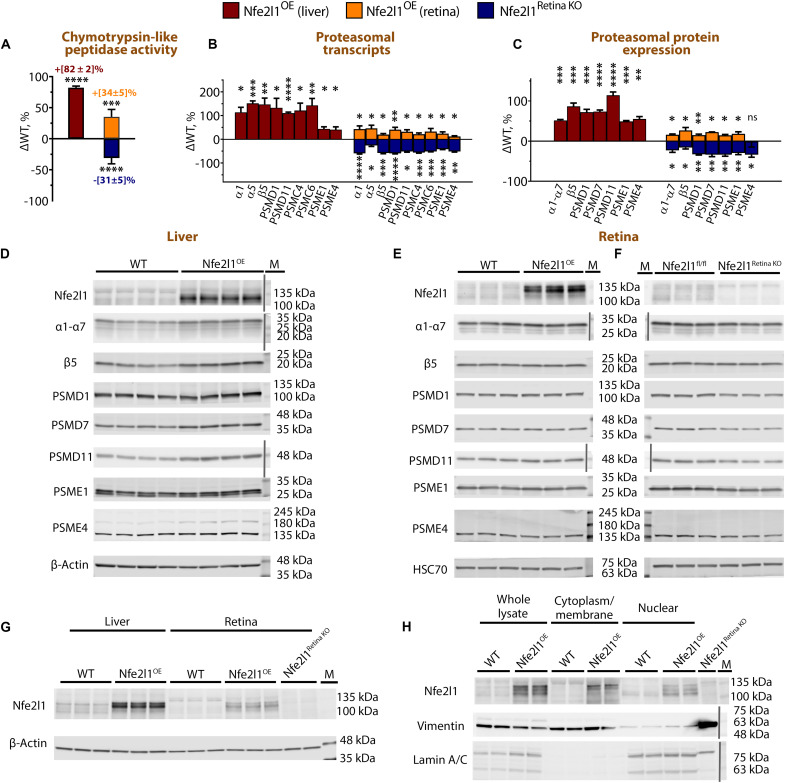
Nfe2l1 levels establish the size of the proteasome pool in retinas. (**A**) Chymotrypsin-like peptidase activity measured in the retinal extracts prepared from Nfe2l1^OE^ and Nfe2l1^Retina KO^ mice and liver extracts prepared from Nfe2l1^OE^ mice. (**B**) Transcriptional analysis of representative proteasome subunits in retinas or livers of the indicated mice performed via RT-qPCR. (**C**) Quantification graphs and (**D** to **F**) Western blots showing proteasomal components detected in (D) livers and (E) and (F) extracts prepared from the retinas of the indicated mice. Changes in proteasome activity, transcript levels, and protein levels were expressed as the percentage of difference (ΔWT) from the average value obtained with WT mice. (**G**) Comparative Western blots showing the levels of Nfe2l1 in the lysates prepared from the livers and retinas of indicated mice. Retinal lysates of Nfe2l1^Retina KO^ mice were used to control for antibody specificity. (**H**) Detection of Nfe2l1 in whole lysates and subcellular fractions prepared from the livers of Nfe2l1-overexpressing mice and their WT littermates. All mice were 1 month old. The data are presented as the mean ± SD. All experiments were repeated at least three times. Color-stained protein markers (M) were either detected as nonspecific bands together with proteins of interest during enhanced chemiluminescence and infrared imaging or added from blot photographs (separated with a vertical gray line).

A fluorogenic chymotrypsin-peptidase assay, a commonly used method to evaluate proteasome activity, showed an 85% higher rate of substrate proteolysis in the livers and a 34% higher rate of substrate proteolysis in the retinas of Nfe2l1^OE^ mice compared to those of wild-type (WT) littermate mice (Fig. 1A). The rate of proteolysis was reduced by 31% in the retinas of the Nfe2l1^Retina KO^ mice (Fig. 1A). Changes in proteasome activity were paralleled by changes in proteasome levels, which were increased in the retinas and livers of the Nfe2l1^OE^ mice and reduced in the retinas of the Nfe2l1^Retina KO^ mice (Fig. 1, B and C). A targeted RT-qPCR (real-time quantitative polymerase chain reaction) transcriptional analysis indicated a 50 to 100% increase in components representative of the 20*S* core particle (α1, α5, β5), 19*S* regulator (PSMD1, PSMD11, PSMC4, and PSMC6), and components of ubiquitin-independent regulators (PSME1 and PSME4) in the livers of the Nfe2l1^OE^ mice (Fig. 1B). In the retinas, the levels of proteasomal components were 5 to 50% higher in the Nfe2l1^OE^ mice and 25 to 50% lower in the Nfe2l1^Retina KO^ mice (Fig. 1B). Transcriptional changes translated into changes in protein levels (Fig. 1, C to F): increased amounts of representative proteasome subunits in the Nfe2l1^OE^ mice (49 to 141% in the livers and 14 to 27% in the retinas) and reduced amounts (a 16 to 34% decrease) in the retinas of the Nfe2l1^Retina KO^ mice.

The detection of basal Nfe2l1 levels in normal unstressed cells and tissues is challenging (*32*). Therefore, the staining of endogenous Nfe2l1 protein from WT mice in the Western blots was weak, but a prominent band was observed for lysates prepared from Nfe2l1^OE^ mice (Fig. 1, D to F). This band was absent in the control retinas of the Nfe2l1^Retina KO^ mice run on the same SDS-PAGE (polyacrylamide gel electrophoresis) gel (Fig. 1, F and G). In Nfe2l1-overexpressing mice, the Western blot bands representing Nfe2l1 in the retinas were less intense than those representing Nfe2l1 in the livers, suggesting a less robust increase, which may explain the smaller effect on proteasomal expression observed in the retinas (Fig. 1G). According to the currently accepted model of Nfe2l1 regulation, Nfe2l1 is an endoplasmic reticulum (ER)–resident protein that undergoes a continuous cycle of synthesis, insertion into the ER membrane, glycosylation/deglycosylation, extrusion through the ER-associated degradation (ERAD) pathway, and proteasomal degradation with a half-life of approximately a few minutes (*33*). The proteolytic products of the Nfe2l1 protein lacking the N-terminal transmembrane domain, when not destroyed by proteasomes, translocate to the nucleus, where they drive the expression of proteasome subunits. The details of the Nfe2l1 protein life cycle, a full list of its regulators, and the number and size of proteolytic products are still under investigation and continuously revised (*33*, *34*). Nevertheless, our data are in agreement with the general picture of Nfe2l1 regulation. On the one hand, overexpression of Nfe2l1 increased the fraction of Nfe2l1 escaping proteasomal degradation and reaching the nucleus, as evident from its enrichment in the nuclear fraction (Fig. 1H). On the other hand, the retinas of Nfe2l1^Retina KO^ mice showed reduced levels of proteasomes, indicating the contribution of basal Nfe2l1 levels to the amounts of proteasomes in retinas (Fig. 1, B, C, and F).

**Fig. 2.**
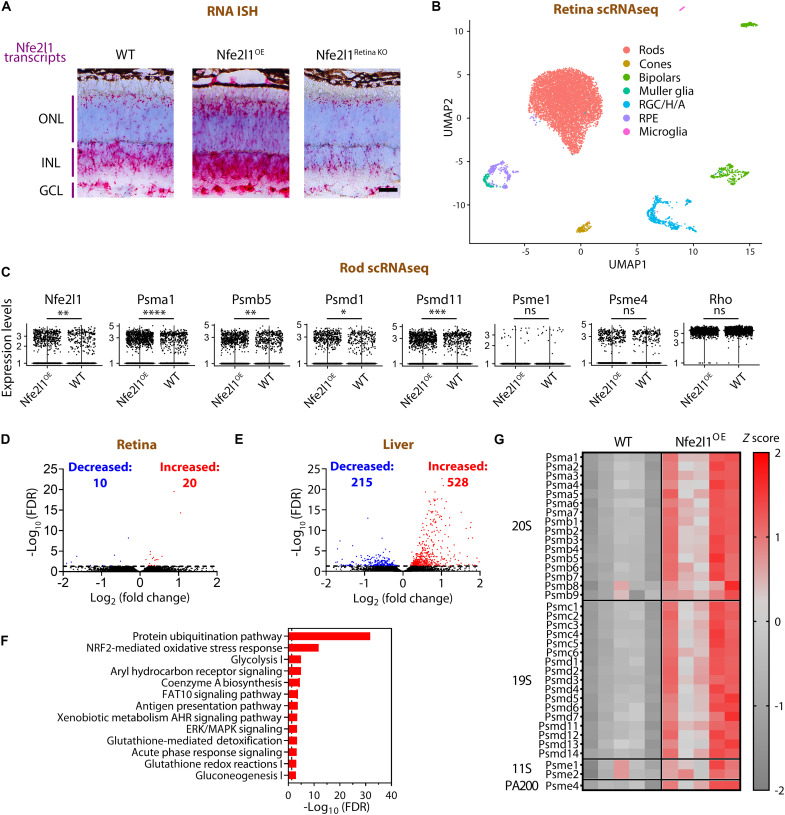
Nfe2l1 overexpression increases the levels of proteasome transcripts in rod photoreceptors. (**A**) Nfe2l1 transcripts detected in the retinas of the indicated mice via RNA in situ hybridization (ISH). ONL, outer nuclear layer (containing photoreceptor nuclei); INL, inner nuclear layer; GCL, ganglion cell layer. Scale bar, 25 μm. See also fig. S1 for retinal cross sections. (**B**) Uniform manifold approximation and projection (UMAP) plot of cells prepared from Nfe2l1^OE^ and WT mouse retinas. (**C**) Expression levels of Nfe2l1 and proteasome transcripts in the rod photoreceptor fractions of the indicated mice. Rhodopsin (Rho) transcripts serve as control markers for rods. (**D** and **E**) Volcano plots showing differentially expressed genes in (D) retinas and (E) livers of Nfe2l1^OE^ mice as detected with bulk RNAseq. Black dots represent genes with a false discovery rate less than 0.05. (**F**) Top pathways affected by Nfe2l1 overexpression in livers and identified with Ingenuity Pathway Analysis software (QIAGEN, Hilden, Germany). See also data S1 (retina) and S2 (liver) for complete lists and analysis. (**G**) Heatmap of changes in proteasome transcripts in livers of Nfe2l1^OE^ and WT mice calculated from raw counts and presented as *z* scores. FDR, false discovery rate.

In the next set of experiments, we sought to confirm an increase in the Nfe2l1 and proteasomal transcripts in rod photoreceptors, the primary cells of interest in our study. An RNA ISH (in situ hybridization) analysis of Nfe2l1^OE^ mice showed a panretinal increase in Nfe2l1 transcript levels in all retinal layers, including increased staining in the outer nuclear layer (ONL) containing photoreceptor nuclei (Fig. 2A, see also fig. S1). The analysis of control retinas from Nfe2l1^Retina KO^ mice showed nearly complete but mosaic loss of Nfe2l1 transcripts (Fig. 2A, see also fig. S1). Next, we used single-cell RNAseq (scRNAseq) and a cell-selective strategy to confirm an increase of proteasome transcripts in rods. As shown in Fig. 2B, we readily distinguish and isolate rod photoreceptors in uniform manifold approximation and projection (UMAP) plots in our single-cell datasets. An increase in Nfe2l1 and proteasome transcripts was evident from the elevated average values and a higher fraction of sequenced rods containing transcripts of interest in Nfe2l1^OE^ mice compared to WT littermates (Fig. 2C, see also table S1 for analysis of other proteasome subunits). Bulk RNAseq of Nfe2l1^OE^ retinas did not show a coordinated increase in proteasome levels (Fig. 2D, see also data S1), indicating that the measure was likely not sensitive enough to identify an ~20 to 30% change, which we detected with targeted RT-qPCR, and confirmed with Western blotting and peptidase measurements as described above. At the same time, an analysis of Nfe2l1^OE^ livers with bulk RNAseq showed a robust transcriptional response (Fig. 2E, see also data S2), with top changes affecting genes belonging to “Protein Ubiquitination Pathway” (Fig. 2F, see also data S2). These transcriptional changes included a coordinated up-regulation of transcripts for proteasomal subunits (Fig. 2G). In the next set of experiments, we showed that ~30% increase in proteasomal activity and Nfe2l1 overexpression was sufficient to stimulate degradation of ubiquitinated proteins in rods of mice stressed by misfolded transmembrane proteins.

### Nfe2l1 overexpression counteracts ubiquitin-proteasome insufficiency in a heterozygote Rho^P23H/WT^ knock-in mouse model of human blindness

Previously, we and other researchers reported that photoreceptor neurons in genetically diverse mouse models of retinal degeneration accumulated the ubiquitin-proteasome reporter Ub^G76V^-GFP (ubiquitin fused with green fluorescent protein and containing glycine to valine amino acid substitution at 76th position), which served as a readout for the degradation of short-lived ubiquitinated proteins (*16*, *35*–*38*). The specific changes in the photoreceptors of these models that interfere with the processing of the Ub^G76V^-GFP reporter, impairing its clearance, are unknown. Similarly, it is unclear whether the rate-limiting steps in processing ubiquitinated proteins in these diverse models are the same. Nevertheless, an accumulation of this reporter can be interpreted to be a manifestation of limited UPS capacity (UPS insufficiency) to degrade proteins, which becomes evident in photoreceptors showing an increased burden of misfolded and mistargeted proteins (*35*). These mice allow us to assess the impact of proteasome increase on protein degradation in vivo.

**Fig. 3.**
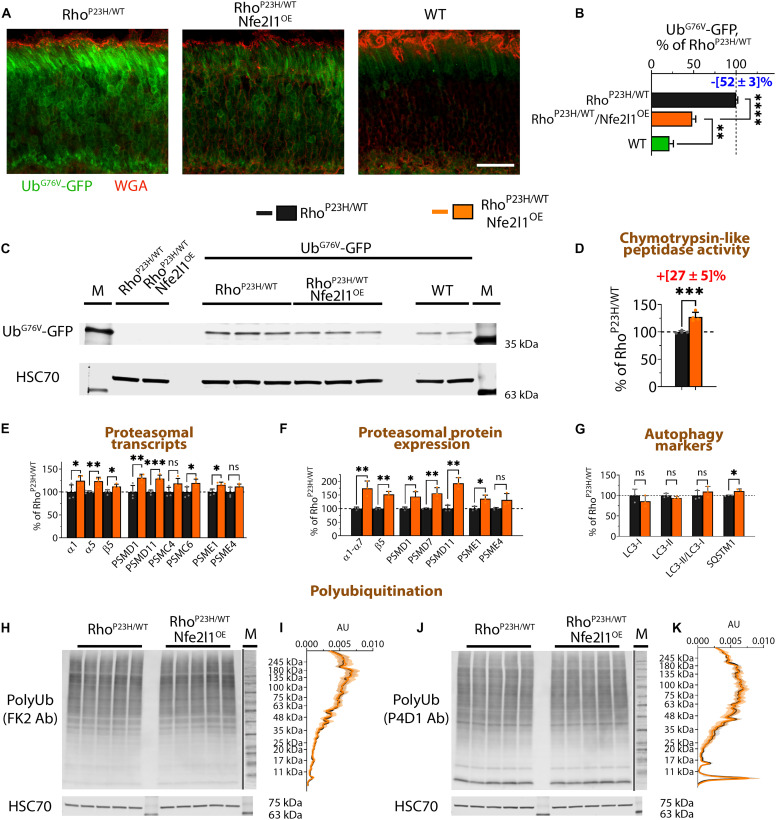
Nfe2l1 overexpression counteracts UPS insufficiency in a Rho^P23H/WT^ mouse model of human blindness. (**A**) Ub^G76V^-GFP reporter (green) in retinal cross sections of Rho^P23H/WT^ and Rho^P23H/WT^/Nfe2l1^OE^ littermates, and Ub^G76V^-GFP/WT control mice. Rod outer segments (red) were stained with wheat germ agglutinin (WGA). Scale bar, 25 μm. (**B**) Quantification plot and (**C**) representative Western blot of the Ub^G76V^-GFP reporter in retinas of mice with the indicated genotypes detected with an anti-GFP antibody. Extracts prepared from littermates negative for the Ub^G76V^-GFP transgene were used to control for antibody specificity. The results are shown as a percentage of the average signal in Rho^P23H/WT^/Ub^G76V^-GFP mice. (**D**) Chymotrypsin-like peptidase activity was measured in the extracts prepared from retinas of Rho^P23H/WT^ and Rho^P23H/WT^/Nfe2l1^OE^ littermate mice. (**E**) Transcription analysis of the representative proteasome subunits in indicated mice was performed with RT-qPCR. Quantification graphs of the Western blot bands for (**F**) proteasome components and (**G**) autophagy markers detected in the extracts prepared with retinas from the indicated mice. Images of Western blots quantified to generate the plots are shown in fig. S2. (**H** to **K**) Polyubiquitin chains in the extracts prepared from retinas of the indicated mice as detected by Western blotting with (H) FK2 and (J) P4D1 antibodies. (I) and (K) Averaged density profiles of the polyubiquitin staining shown in (H) and (J). All animals were 28 days old. The data are presented as the mean ± SD (B) and (D) to (G) or the mean ± 95% CI (I) and (K).

A heterozygote Rho^P23H/WT^ knock-in mouse is an established model of human blindness called retinitis pigmentosa (*39*, *40*), expressing both mutant and WT rhodopsin in rod photoreceptors. A proline-to-histidine (P23H) amino acid substitution at 23rd position of rhodopsin destabilized the structure of the transmembrane protein rhodopsin, driving its constant ERAD-associated polyubiquitination and proteasomal degradation, which stresses rod photoreceptors, eventually leading to their death (*39*, *40*). As shown in Fig. 3A and described in our previous studies, we observed an accumulation of Ub^G76V^-GFP reporter in the photoreceptors of these mice. Therefore, Rho^P23H/WT^ mouse line expressing the Ub^G76V^-GFP reporter might serve as an efficient model to examine methodologies to modulate the efficiency of UPS functioning in vivo (*16*, *24*).

As shown in Fig. 3A, overexpression of Nfe2l1 improved clearance of Ub^G76V^-GFP reporter in photoreceptors of Rho^P23H/WT^ mice detected with confocal microscopy. In agreement with the imaging findings, Western blot (Fig. 3, B and C) quantification showed an approximately twofold reduction in steady-state reporter level. Control experiments confirmed the increase in proteasome activity and higher levels of proteasomes in Rho^P23H/WT^/Nfe2l1^OE^ retinas (Fig. 3, D to F), similar to the changes described for the Nfe2l1^OE^ mice above. Quantitative Western blotting did not detect changes in autophagy markers, LC3-I and LC3-II (microtubule-associated proteins 1A/1B light chain 3B-I/II), or their ratios but showed a slight increase in the levels of SQSTM1 (sequestosome-1) protein (Fig. 3G). The Western blot of ubiquitination patterns in retinal lysates probed with FK2 antibody generated against polyubiquitinated chains (Fig. 3, H and I) and P4D1 antibody raised against monoubiquitin (Fig. 3, J and K) did not reveal a difference between Rho^P23H/WT^/Nfe2l1^OE^ and Rho^P23H/WT^ littermate mice. Similarly, the ubiquitination patterns in the extracts prepared from the retinas of Nfe2l1^OE^ mice and WT littermates were undistinguishable (fig. S2). Therefore, Nfe2l1 overexpression does not affect the steady-state levels of polyubiquitinated proteins as detected with Western blotting. Yet, better clearance of reporter in combination with elevated proteasomal activity suggests some extent of improvement in UPS functioning in stressed rods, at least with the processing of some protein substrates. According to the current concept in the field, it is thought that mutant P23H rhodopsin efficiently and almost entirely degraded, with a small fraction of rhodopsin immunoprecipitated from retinas of Rho^P23H/WT^ mice found to be modified with ubiquitin (*39*, *40*). In our analysis, we did not detect differences in the total levels of rhodopsin and ubiquitinated rhodopsin (fig. S2) in fractions enriched for polyubiquitin (using polyubiquitin binding domains) or rhodopsin (using anti-rhodopsin antibody) in Rho^P23H/WT^/Nfe2l1^OE^ mice. Thus, Nfe2l1 overexpression does not appear to complement already efficient rhodopsin degradation, at least as could be assessed by levels of ubiquitinated rhodopsin with available tools. Nevertheless, a more effective clearance of reporter points to the benefits of Nfe2l1 overexpression and an improvement in UPS functioning, e.g., aiding with degradation of an everyday basal load of damaged, misfolded, or mistranslated proteins in already stressed Rho^P23H/WT^ rods. In the experiments described in the next section, we applied in vivo imaging and physiological methods to show quantitatively that Nfe2l1 overexpression and higher proteasomal activity slowed down vision loss in Rho^P23H/WT^ mice.

### Nfe2l1 overexpression delays vision loss in a heterozygote Rho^P23H/WT^ knock-in model of human blindness

**Fig. 4.**
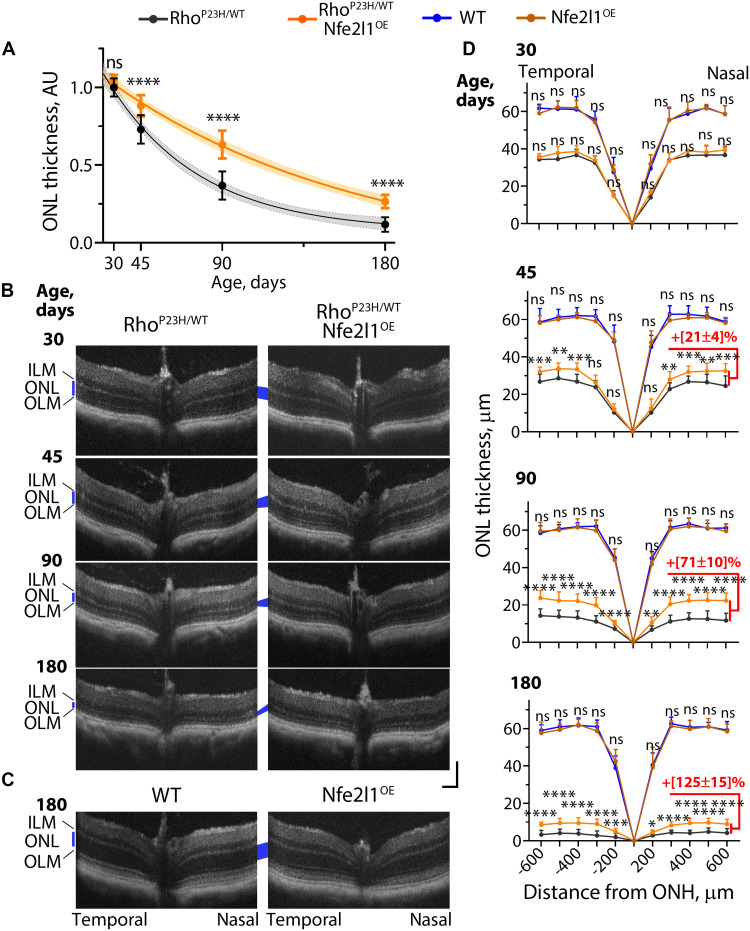
Nfe2l1 overexpression delays retinal degeneration in a Rho^P23H/WT^ mouse model of human blindness. (**A**) Comparative analysis of age-related thinning of the ONL in Rho^P23H/WT^ and Rho^P23H/WT^/Nfe2l1^OE^ mice. To generate the plot, the measurements from horizontal optical coherence tomography (OCT)–based spider diagrams built around optic nerve head (ONH) at the indicated ages were summed, normalized to average values of 30-day-old Rho^P23H/WT^ mice, and fitted with an exponent. (**B** and **C**) Representative horizontal SD-OCT scans and (**D**) OCT-based spider diagrams showing ONL thickness at indicated ages. The ONL is marked with a blue quadrilateral. The scale bar for the OCT images is 100 μm. The numbers of eyes analyzed at P30 were as follows: Rho^P23H/WT^—8, Rho^P23H/WT^/Nfe2l1^OE^—5, WT—4, Nfe2l1^OE^—3; at P45: Rho^P23H/WT^—14, Rho^P23H/WT^/Nfe2l1^OE^—12, WT—8, Nfe2l1^OE^—8; at P90: Rho^P23H/WT^—14, Rho^P23H/WT^/Nfe2l1^OE^—12, WT—10, Nfe2l1^OE^—22; at P180: Rho^P23H/WT^—14, Rho^P23H/WT^/Nfe2l1^OE^—12, WT—11, Nfe2l1^OE^—13. The data are presented as the mean ± SD. Quantification was performed by individuals not aware of genotype.

**Fig. 5.**
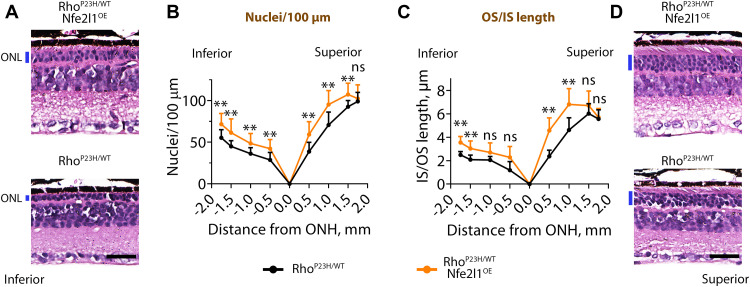
Nfe2l1 overexpression improves photoreceptor survival in a Rho^P23H/WT^ mouse model of human blindness. Morphometric analysis of retinas obtained from 6-month-old mice of the indicated genotypes. (**A** and **D**) Images of the representative regions of H&E-stained retinal cross sections from (A) inferior and (D) superior parts of the retinas ~750 μm from the center of the ONH. Scale bar, 25 μm. (**B**) Spider diagrams show the number of photoreceptor nuclei in 100-μm segments counted along the inferior-superior axis of the mouse eyes and (**C**) the distance from the outer limiting membrane to the tip of the outer segments (IS/OS length) measured at the indicated distances from the center of the ONH. The representative cross sections cut through an entire retina are shown in fig. S3. The number of eyes analyzed was as follows: Rho^P23H/WT^—10 and Rho^P23H/WT^/Nfe2l1^OE^—13. The data are presented as the mean ± SD. Quantification was performed by individuals not aware of genotype.

Optical coherence tomography (OCT) allows efficient quantitative in vivo studies of retinal structures (*41*). As shown in Fig. 4 (A and B), the ONL, containing photoreceptor nuclei, in the Rho^P23H/WT^ mice became progressively thinner with age (marked in blue) and was barely discernible after the mice reached 6 months of age. Overexpression of Nfe2l1 delayed ONL thinning in the Rho^P23H/WT^ mice (Fig. 4, A to C). The ONL thickness around the optic nerve head (ONH) in Rho^P23H/WT^/Nfe2l1^OE^ mice, presented in the form of spider diagrams, was consistently thicker compared to that in Rho^P23H/WT^ littermates older than 45 days (Fig. 4D). A morphometric analysis confirmed an increase in photoreceptor survival in Nfe2l1^OE^-overexpressing animals (Fig. 5). We analyzed retinal cross sections containing ONH and cut along the superior-inferior line of eye (fig. S3). Samples prepared from 6-month-old Rho^P23H/WT^/Nfe2l1^OE^ mice showed from one to three (Fig. 5B) additional rows of surviving photoreceptor nuclei and 10 to 90% longer outer segments (Fig. 5C) throughout the entire retina compared to those in the Rho^P23H/WT^ littermate mice. OCT (Fig. 4, C and D) studies and morphometric analysis (fig. S3) did not identify long-term adverse effects of Nfe2l1 overexpression: The retinal structure and the number of nuclei in the Nfe2l1^OE^ mice were indistinguishable from those in the WT littermates. In the next set of experiments, we showed that better photoreceptor preservation in the Rho^P23H/WT^/Nfe2l1^OE^ mice translated into improved retinal function.

**Fig. 6.**
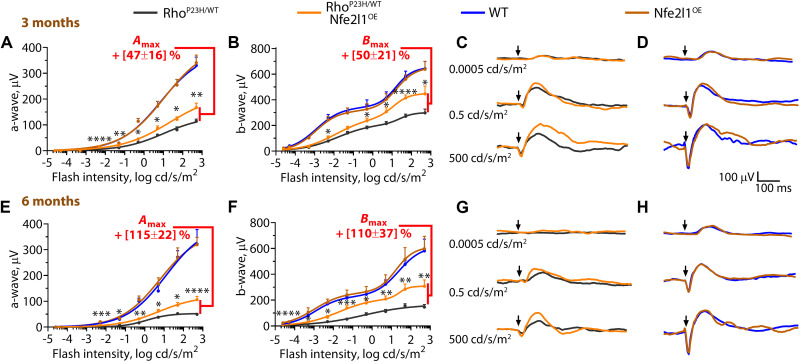
Nfe2l1 overexpression delays vision loss in a Rho^P23H/WT^ mouse model of human blindness. Response amplitudes of electroretinography (ERG) a- and b-waves evoked by light flashes of increasing intensity in the mice with the indicated genotypes as determined at (**A** to **D**) 3 and (**E** to **H**) 6 months of age. The number of eyes analyzed at 3 months was as follows: Rho^P23H/WT^—13, Rho^P23H/WT^/Nfe2l1^OE^—12, WT—7 eyes, and Nfe2l1^OE^—7. The number of eyes analyzed at 6 months was as follows: Rho^P23H/WT^—12, Rho^P23H/WT^/Nfe2l1^OE^—12, WT—6, Nfe2l1^OE^—12. (C), (D), (G), and (H) Representative ERG recordings evoked by flashes of indicated light intensities. The data are presented as the mean ± SEM.

Electroretinography (ERG) is a physiological method for quantitatively assessing retinal function in vivo (*42*). In typical ERG studies, dark-adapted mice are exposed to bright flashes with increasingly intense light to assess scotopic (rod), mesopic (mixed rod and cone), and photopic (cone) vision. In ERG traces, the a-wave responses originate from rod and cone photoreceptors, and the b-wave represents amplified responses from retinal neurons downstream of photoreceptors. The amplitudes of both waves are effective quantitative measures of the number and health of surviving photoreceptors. The ERG recordings of Rho^P23H/WT^/Nfe2l1^OE^ mice consistently yielded higher responses, with the maximum a- and b-wave amplitudes recorded to be 50 and 100% higher in 3- and 6-month-old animals compared to those in Rho^P23H/WT^ littermates (Fig. 6, A to C and E to G). ERG responses of Nfe2l1^OE^ mice and their WT littermates were indistinguishable from each other (Fig. 6, A, B, D to F, and H).

An OCT analysis (Fig. 4A) showed that Nfe2l1 overexpression delayed vision loss in Rho^P23H/WT^ mice for approximately 2 months. Notably, this form of retinal degeneration progresses slower in humans than in mice (years instead of months) (*39*). Therefore, months of delayed photoreceptor loss in mice might translate into years of preserved vision in humans. Furthermore, here, we focused only on target validation, and the extent of the observed treatment might be limited by the characteristics of the available Nfe2l1^OE^ transgenic mouse line. A more efficient approach to augment the Nfe2l1 pathway may potentially lead to greater improvement.

### Nfe2l1 overexpression and genetic activation of mTORC1 counteract UPS insufficiency and drive proteasome biogenesis in rods stressed with misfolded cytosolic proteins

Ongoing pathological changes in photoreceptors and the nature of proteotoxic stress can reduce the efficiency of Nfe2l1 activation, thus limiting the extent of the proteasome increase. Therefore, in a final set of experiments, we sought to generalize our findings and examine the modulation of proteasome biogenesis in an alternative model of photoreceptor degeneration caused by misfolded cytosolic protein–induced stress. In Gγ_1_^−/−^ mice (mice lacking *G protein subunit gamma transducin 1* gene), the misfolded G protein subunit beta 1 (Gβ_1_ subunit hereafter) could not be stabilized without its functional partner, the Gγ_1_ subunit. Continuous degradation of misfolded Gβ_1_ subunit overloads UPS in rod photoreceptors, until they die (*35*). Consistent with this model, we previously found that the Ub^G76V^-GFP reporter accumulated in the rods of Gγ_1_^−/−^ mice (*35*). This form of photoreceptor degeneration is different from that in Rho^P23H/WT^ mice, which is caused by stress induced by misfolded transmembrane protein rhodopsin continuously degraded via ERAD pathway (*40*). As discussed below, we not only probed the effectiveness of Nfe2l1 overexpression to stimulate proteasome biogenesis and counteract UPS insufficiency in Gγ_1_^−/−^ mice but also compared effects with those caused by genetic activation of mTORC1. We crossed Gγ_1_^−/−^ and Nfe2l1-overexpressing mice to generate the Gγ_1_^−/−^/Nfe2l1^OE^ mouse line. Genetic activation of the mTORC1 pathway was achieved by deleting its negative regulator Tsc2 specifically in the rods of the Gγ_1_^−/−^ mice (hereafter, the Gγ_1_^−/−^/Tsc2^Rod KO^ mouse line).

**Fig. 7.**
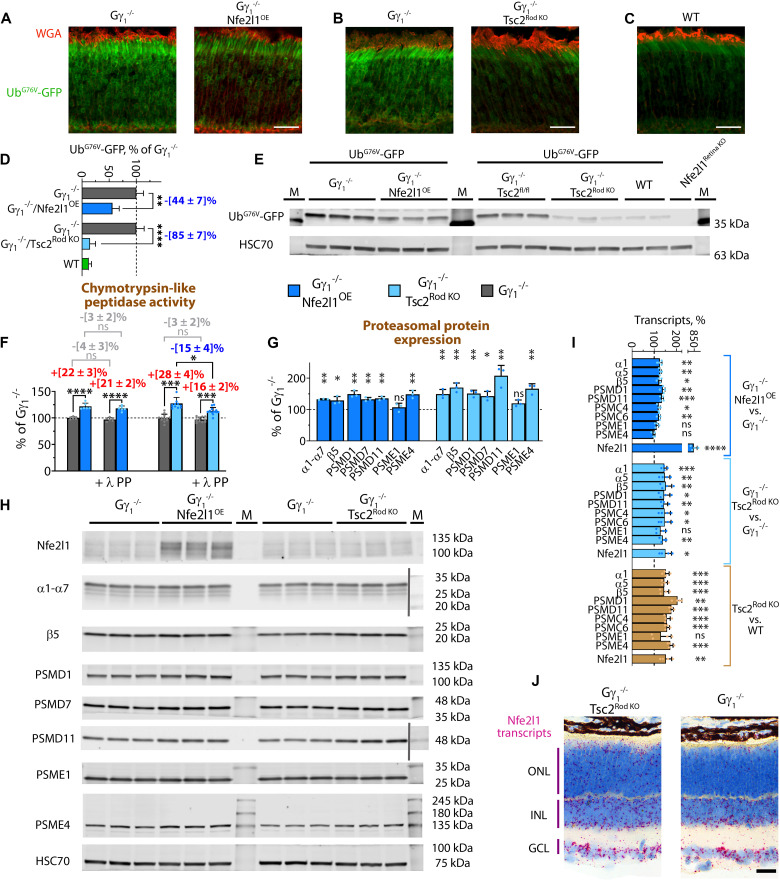
Nfe2l1 overexpression and Tsc2 knockout counteract UPS insufficiency in a Gγ_1_^−/−^ mouse model of photoreceptor degeneration. (**A** to **C**) Fluorescence signal of Ub^G76V^-GFP reporter (green) in retinal cross-sections of (A) Gγ_1_^−/−^/Nfe2l1^OE^ and (B) Gγ_1_^−/−^/Tsc2^Rod KO^ mice shown along with their Gγ_1_^−/−^ littermates and (C) Ub^G76V^-GFP/WT control mice. The outer rod segments (red) are stained with WGA. Scale bar, 25 μm. (**D**) Quantification plot and (**E**) representative Western blot of the Ub^G76V^-GFP reporter in lysates prepared from the retinas of indicated mice as detected with an anti-GFP antibody. (**F**) Chymotrypsin-like proteasome activity was measured in retinal extracts with or without lambda protein phosphatase treatment (λ PP). (**G**) Quantification plot and (**H**) Western blots showing representative proteasome subunits in the retinal extracts of the indicated mice. The protein markers (M) were detected as nonspecific bands together with proteins of interest or added from blot photographs and separated with a vertical gray line. (**I**) Transcript analysis of Nfe2l1 and representative proteasome subunits in retinas from the indicated mice performed with RT-qPCR and shown as a percentage of the average values for Gγ_1_^−/−^ or WT littermates. (**J**) Nfe2l1 transcripts in the retinas of indicated mice as detected with RNA ISH. See also fig. S4. Scale bar, 25 μm. All animals were 1 month old. The data are shown as the mean ± SD.

As shown in Fig. 7 (A to C), both the overexpression of Nfe2l1 and chronic mTORC1 activation improved clearance of Ub^G76V^-GFP reporter in Gγ_1_^−/−^ photoreceptors. Western blot quantification revealed that the level of the Ub^G76V^-GFP reporter in lysates prepared with retinas of Gγ_1_^−/−^/Nfe2l1^OE^ and Gγ_1_^−/−^/Tsc2^Rod KO^ mice was reduced by 44% and 85%, respectively (Fig. 7, D and E). Chymotrypsin-like peptidase assay showed higher rates of substrate proteolysis in the retinas of both mouse lines, a 22% increase in Gγ_1_^−/−^/Nfe2l1^OE^ and a 28% increase in Gγ_1_^−/−^/Tsc2^Rod KO^ mice compared to Gγ_1_^−/−^ littermates (Fig. 7F). We previously reported that mTORC1-mediated proteasomal activity in the retinas of rod-specific Tsc2 knockout mice, to some extent, was phosphorylation dependent (*24*). Therefore, we studied the impact of lambda protein phosphatase treatment (λ PP) on proteasome activity. Phosphatase treatment slightly reduced proteasome activity (3 to 4%) in the retinal lysates prepared from Gγ_1_^−/−^ and Nfe2l1-overexpressing Gγ_1_^−/−^ littermate mice (Fig. 7F), but this trend did not reach a level of statistical significance. In Gγ_1_^−/−^/Tsc2^Rod KO^ mice, phosphatase treatment decreased proteolytic activity by 15%. Nevertheless, even after treatment, the proteolytic activity remained ~16% higher than that in phosphatase-treated Gγ_1_^−/−^ littermates (Fig. 7F). Quantitative Western blotting demonstrated higher levels of proteasomes in the retinas of both Gγ_1_^−/−^/Nfe2l1^OE^ and Gγ_1_^−/−^/Tsc2^Rod KO^ mice, with a slightly higher effect after mTORC1 activation, particularly for the PSMD11 and β5 proteasome subunits (Fig. 7, G and H). Notably, changes in proteasome expression and activity in Gγ_1_^−/−^/Tsc2^Rod KO^ were most likely low-end estimates since they were measured in whole-retina lysates, but Tsc2 was removed only from rod photoreceptors.

Western blotting for the Nfe2l1 protein in the retinas of Gγ_1_^−/−^ mice produced a weak signal, with a prominent band shown for the Nfe2l1^OE^ mice, and a slight elevation in Gγ_1_^−/−^/Tsc2^Rod KO^ animals (Fig. 7H). Because of complex posttranslational modifications and weak signal, detecting subtle changes in Nfe2l1 levels in the retina by Western blotting is challenging (Fig. 7H). Therefore, we performed a targeted RT-qPCR analysis to confirm the elevation in Nfe2l1 in the Gγ_1_^−/−^/Tsc2^Rod KO^ mice (Fig. 7I). We used RNA ISH to confirm the mTORC1-mediated increase in the number of Nfe2l1 transcripts in the ONL (which contains mostly rod photoreceptor nuclei) of Gγ_1_^−/−^/Tsc2^Rod KO^ mice as an additional control (Fig. 7J, see also fig. S4). The mTORC1-mediated increase of proteasome transcripts in the Gγ_1_^−/−^/Tsc2^Rod KO^ and Tsc2^Rod KO^ mice was comparable (Fig. 7I). This observation contrasts our previous findings for degenerating retinas of Rho^P23H/WT^/Tsc2^Rod KO^ mice stressed by misfolded transmembrane P23H mutant protein, in which this transcriptional response was suppressed (*24*). Therefore, the type of proteotoxic stress and form of retinal degeneration may affect the efficiency of Nfe2l1 activation, particularly mediated via chronic stimulation of mTORC1 pathway. Thus, our analysis showed that in retinas of Gγ_1_^−/−^ mice stressed by misfolded cytosolic protein, both Nfe2l1 overexpression and chronic mTORC1 activation were efficient in driving proteasome biogenesis and counteracting UPS insufficiency. However, whereas in Nfe2l1- overexpressing mice higher proteasomal activity could be attributed to an increase in proteasomal amounts, chronic stimulation of mTORC1 pathway could augment proteasomal capacity through a combination of increasing proteasomal pool and phosphorylation-mediated stimulation of proteasomes. Notably, this in vivo comparison might point to natural limits (~30%) to which proteasomal activity could be enhanced through either mechanism in the retina. Future studies might have to consider more potent combinatorial approaches to stimulate proteasomal degradation.

## DISCUSSION

Dysregulated proteostasis is a hallmark of many inherited and age-related human diseases, including retinal degeneration. The approaches allowing improved efficiency to clear misfolded proteins could be applied to treat a broad range of human diseases. Mouse models of photoreceptor degeneration stressed by misfolded proteins and accumulating UPS reporter could serve as effective tools for proof-of-principle studies and testing approaches to manipulate proteostasis, particularly since the outcomes of these treatments on photoreceptor survival can be monitored quantitatively using modern in vivo imaging and physiological techniques. Our study shows that overexpression of Nfe2l1 does not have adverse effects on retinal function or structure, drives proteasomal activity and expression, improves clearance of UPS reporter in photoreceptors struggling with misfolded proteins, and delays vision loss in a mouse model of human blindness. It is worth noting that an assessment of the benefits of Nfe2l1 overexpression is limited by the characteristics of the Nfe2l1^OE^ transgenic mouse line. A more efficient approach to augment the Nfe2l1 pathway may lead to even better photoreceptor survival, but we cannot exclude the possibility that overactivation of the Nfe2l1 pathway might also be detrimental. Our findings indicate that, to some extent, an elevated proteasomal activity (~30%) could be tolerated without cellular pathology and even be beneficial under certain stressors. The findings pave the way to consider the relatively poorly investigated Nfe2l1 pathway as a therapeutic target for treating neurodegenerative diseases linked to protein misfolding and promote drug development to enhance its activity.

An overexpression of transcriptional factor might have pleiotropic effects, making it difficult to pinpoint with certainty mechanisms leading to the improved survival of photoreceptors. Nfe2l1 has become a topic of increasing interest in recent years. It was proposed to play a role in controlling proteasomal levels and other genes involved in proteostasis regulation and protection against oxidative stress and serve as a cholesterol and metabolic sensor and regulator of ferroptosis (*29*, *33*, *43*–*48*). In the livers of Nfe2l1^OE^ mice, in addition to the higher levels of proteasomes, we observed transcriptional changes in genes involved in protein degradation, regulation of glucose and redox metabolism, and oxidative stress response. Our RNAseq analysis of Nfe2l1^OE^ retinas did not identify similar marked transcriptional rearrangements, potentially due to the lower extent of Nfe2l1 up-regulation and less efficient Nfe2l1 activation in comparison to the liver. Yet, we cannot exclude the possibility that retinal improvement in Rho^P23H/WT^/Nfe2l1^OE^ mice is not exclusively attributed to the changes in proteasome amounts but also caused by slight transcriptional changes affecting other genes and Nfe2l1-mediated signaling pathways. Still, to date, the most clearly documented and understood function of Nfe2l1 is to control the levels of proteasomes and drive an increase in proteasome transcripts in response to sublethal doses of proteasome inhibitors (*49*, *50*). Our findings warrant studies of the Nfe2l1 pathway in the retina and focus on its enhanced activation for the treatment of diseases associated with protein misfolding, including drug development. In this regard, a recent large high-throughput drug screening for Nfe2l1 activators did not lead to the identification of potential candidates, except for molecules that augmented proteasome transcription triggered via proteasomal inhibition (*51*). Our comparison of mice with genetic activation of the mTORC1 pathway and mice overexpressing Nfe2l1 predicts that sustained up-regulation of proteasome transcription and activity achieved through Nfe2l1 augmentation in vivo may be relatively small (~30%). Therefore, the use of sensitive methods allowing the detection of small changes in proteasome expression and activity might be essential for the identification of potent Nfe2l1 activators by drug screening. At the same time, the findings are also encouraging since even a 30% increase in proteasomal levels and activity might be sufficient to produce therapeutic effects.

A side-by-side comparison of proteasomal biogenesis in the retinas of Gγ_1_^−/−^ mice with activated mTORC1 pathway and mice overexpressing Nfe2l1 clearly showed that in vivo mTORC1 activation is as efficient, if not more efficient, in driving proteasome biogenesis without substantial Nfe2l1 increase (at least in comparison to Nfe2l1-overexpressing mice). This observation highlights and encourages molecular studies of poorly defined mechanisms underlying mTORC1-driven Nfe2l1 activation, which could lead to the identification of more targeted and potent approaches to stimulate the Nfe2l1 pathway (*52*).

Finally, our findings add Nfe2l1 to the growing list of transcriptional master regulators of proteostasis that are being investigated as potential targets to delay vision loss. A series of studies solidified the critical role of basal activity of ubiquitously expressed activating transcription factor 6 (ATF6) in regulating stress responses of rods in retinitis pigmentosa and the preservation of cones in humans (*53*–*56*). Recently, modulation of autophagy through selective manipulation of the retinoic acid receptor alpha (RARα) transcriptional program has been shown to increase photoreceptor survival in the retinal degeneration 10 (rd10) mouse model of retinitis pigmentosa (*57*). Together with our findings, these previous studies support the mapping and analysis of transcription networks controlling proteostasis in the retina to harness the potential of these factors to treat age-related and inherited retinal degeneration in a gene- and mutation-independent manner.

## MATERIALS AND METHODS

### Animals and animal procedures

Transgenic mice overexpressing Nfe2l1 (Nfe2l1^OE^, MGI:5804124) were recovered using frozen sperm (RBRC10149) purchased from RIKEN BioResource Research Center (Kyoto, Japan) and were previously described in (*30*). Transgenic mice expressing Ub^G76V^-GFP and Gγ_1_^−/−^ mice were previously described in (*58*, *59*). The Rho^P23H/P23H^ mice were purchased from The Jackson Laboratory (stock #005105). Mice with floxed (Nfe2l1^fl/fl^) fifth exon of *Nfe2l1* gene were generated at Cyagen (Santa Clara, CA, USA) using previously published genetic strategy (*45*). *Chx10-Cre* mice expressing Cre in retina were previously described in (*31*). Tsc2^Rod KO^ mice were derived by crossing Tsc2^fl/fl^ (The Jackson Laboratory, stock #027458) and iCre75 (The Jackson Laboratory, stock #015850) mouse lines and were previously described in (*24*, *60*). Rhodopsin knockout mice were previously described in (*61*). Breeding schemes for all mouse lines and littermates used in experiments are indicated in table S2. Animals were reared under a normal day/night cycle and handled according to the protocols approved by the Institutional Animal Care and Use Committee of the University of Florida (#202009934). Littermates of both sexes were used and processed as a group. Mouse genotypes were determined using real-time PCR with specific probes designed for each gene (Transnetyx, Memphis, TN, USA). All lines were tested negative for retinal degeneration 1 (rd1) and 8 (rd8) mutations. All experiments were performed using littermate controls. Noninvasive experiments with animals (ERG and OCT) were performed as previously described (*24*).

### Western blotting, proteasome activity assays, and polyubiquitin and rhodopsin enrichment assays

Western blotting was performed using previously described protocols with antibodies listed in table S3 (*24*). Retina or liver tissue was sonicated in radioimmunoprecipitation assay (RIPA) lysis buffer (20-188, EMD Millipore, Burlington, MA, USA), supplemented with a Halt Protease or Halt Protease and Phosphatase (78429 and 78440, Thermo Fisher Scientific, Waltham, MA, USA) inhibitor cocktails. The samples used for Western blotting of polyubiquitinated chains were prepared in the presence of 5 μM PR-619 (Life Sensors, Malvern, PA, USA) inhibitor of deubiquitinases/deubiquitylases/ubiquitin-like isopeptidases. The total protein concentration was measured using the Pierce 660 nm Protein Assay Reagent (22660, Thermo Fisher Scientific). Samples were brought to the same concentration in Laemmli Buffer [50 mM tris-HCl (pH 6.8), 2% SDS, 20 mM dithiothreitol (DTT), 10% glycerol, 0.01% bromophenol blue], heated at 95°C for 5 min, and immediately used for experiments. Samples containing 35 to 50 μg of total protein were resolved on a precast 4 to 20% tris-glycine gel (5678094, Bio-Rad Laboratories, Hercules, CA, USA) and transferred to a 0.45-μm PVDF (polyvinylidene fluoride) membrane (IPFL00010, EMD Millipore, Burlington, MA, USA) using wet transfer. Detailed information for blocking methods, antibody dilutions, and detection methods are listed in table S3. Protein bands were visualized with the Odyssey Infrared Imaging System (LI-COR Biosciences, Lincoln, NE, USA) or ChemiDoc Imaging System (Bio-Rad Laboratories, Hercules, CA, USA). Western blots were quantified in ImageJ software using rectangle tool: On each quantified blot, the individual bands/lanes of the same size were selected. The measurements were normalized on values obtained for housekeeping proteins (β-actin or HSC70). The linear range of detection was established in pilot experiments with serial dilutions of samples, adjustments of antibody concentrations, and imaging settings.

Chymotrypsin-peptidase assay was performed as previously described (*24*). Subcellular fractionation was performed on fresh liver tissue (~100 mg from each mouse) using NE-PER Nuclear and Cytoplasmic Extraction Reagents (78833, Thermo Fisher Scientific, Waltham, MA, USA) following the manufacturer’s instructions. Lamin A/C and vimentin were used as markers for nuclei and cytosolic/membrane fractions. Polyubiquitinated proteins were enriched using TUBE2 (tandem ubiquitin binding entities 2) polyubiquitin binding protein domains bound to magnetic beads (UM-402M, Life Sensors, Malvern, PA, USA). Lysates prepared from Rho^−/−^ retinas were used to control for anti-rhodopsin antibody specificity and from Rho^P23H/P23H^ mice as samples lacking WT rhodopsin. Retinas were disrupted in Dounce tissue homogenizer and cleared by centrifugation at 14,000*g* for 10 min under refrigeration. Lysates were brought to the same protein concentration and rotated with TUBE2 beads for 2 hours in the cold room (~200 μg of total protein/30 μl of beads). Following incubation, the beads were washed three times, and captured proteins were eluted by heating beads in 100 μl of Laemmli Buffer at 95°C for 5 min. Aliquots containing input and flowthrough fractions (15 μg of protein) and 20 μl of eluates were used for Western blot analysis with anti-polyubiquitin (FK2) and anti-rhodopsin (1D4) antibodies. For rhodopsin immunoprecipitation, retinal lysates (~200 μg of total protein) were incubated for 2 hours in the cold room with 5 μg of anti-rhodopsin 1D4 antibody preincubated with 25 μl of Dynabeads (10003D, Invitrogen, Waltham, MA, USA) following the manufacturer’s instructions. Rhodopsin was eluted by heating beads in 100 μl of Laemmli Buffer (95°C for 10 min), and 20 μl of eluates was used for Western blot analysis with anti-polyubiquitin (FK2) or anti-rhodopsin (B630) antibodies. The lysates for rhodopsin or polyubiquitin enrichment were prepared in the Lysis Buffer [50 mM tris-HCl (pH 7.5), 0.15 M NaCl, 1 mM EDTA, 1% NP-40, 10% glycerol] supplemented with protease/phosphatase/deubiquitinase inhibitors described above, and the TBST buffer [20 mM tris-HCl (pH 8.0), 0.15 M NaCl, 0.1% Tween 20] was used to wash beads.

### Transcriptomic analysis and RNA ISH

Bulk RNAseq of retinas and differentially expressed gene analysis was performed as previously described in (*62*). For bulk RNAseq transcriptomic studies, the total RNA was prepared from the retinas and livers of five Nfe2l1^OE^ (four males and one female) and WT (four males and one female) littermate mice of 6 to 7 weeks of age. Differentially expressed genes are shown in Microsoft Excel files data S1 (retina) and S2 (liver). RT-qPCR was performed using primers listed in table S4 as previously described (*24*). RNA ISH was performed on 5-μm–thick paraffin sections prepared from formalin-fixed eyes with RNAscope probes (Advanced Cell Diagnostics, Hayward, CA, USA) listed in table S5 on the automated Leica Bond platform (Leica Microsystems GmbH, Wetzlar, Germany) following the manufacturer’s instructions. The single-cell libraries were prepared with pooled retinas from one male and one female littermate mice (Nfe2l1^OE^ and WT) using 10x Chromium Platform and following protocols described in (*63*). Sequencing was performed at UF Interdisciplinary Center for Biotechnology Research, and single-cell data analysis was performed as described in our previous studies (*62*, *64*).

### Histology and microscopy

Histology analysis was performed on 5-μm–thick paraffin sections cut through the superior-inferior line of the eye and containing optic nerve, stained with hematoxylin and eosin stain (H&E), and quantified as described in (*24*). An accumulation of the Ub^G76V^-GFP reporter was assessed in 20-μm–thick frozen retinal sections prepared from the eyes fixed in 4% paraformaldehyde phosphate-buffered saline (PBS) solution (*65*). Rod outer segments were stained with WGA (wheat germ agglutinin; Alexa Fluor 555 conjugate, W32464, Thermo Fisher Scientific). Samples for littermate mice were processed together and imaged on a Leica TCS SP8 confocal microscope using the same settings.

All proteasomal assays, Western blotting, RT-qPCR, RNA ISH, and microscopy experiments describe biological replicates and are representative of three and more independently performed experiments on separate groups of mice. The number of independent biological samples analyzed with OCT, ERG, and H&E is shown in the corresponding figure legends.

### Statistical analysis

Statistical differences were considered significant when *P* < 0.05, as determined by the two-tailed Student’s test using GraphPad Software. Differential gene expression analysis of rod scRNAseq datasets was performed using function FindMarkers from Seurat package. *P* values in the figures are indicated as follows: *P < 0.05, **P < 0.01, ***P < 0.001, ****P < 0.0001, and ns, for *P* > 0.05.

## Notes

View/request a protocol for this paper from *Bio-protocol*.
