# Supplementary material for: Overexpression of Nfe2l1 increases proteasome activity and delays vision loss in a preclinical model of human blindness
Source: Sci Adv. 2023 Jul 14;9(28):eadd5479. doi: 10.1126/sciadv.add5479 (PMC10348684; doi:10.1126/sciadv.add5479)
Supplement: Supplementary file 1 — Figs. S1 to S4 Tables S1 to S5 Legends for data S1 and S2 [file sciadv.add5479_sm.pdf]

Science Advances
AAAS

Supplementary Materials for

Overexpression of Nfe2l1 increases proteasome activity and delays vision loss in a preclinical model of human blindness

Yixiao Wang *et al.*

Corresponding author: Ekaterina S. Lobanova, elobanova@ufl.edu

Sci. Adv. **9**, eadd5479 (2023)
DOI: 10.1126/sciadv.add5479

The PDF file includes:

Figs. S1 to S4
Tables S1 to S5
Legends for data S1 and S2

Other Supplementary Material for this manuscript includes the following:

Data S1 and S2

Fig. S1. Nfe2l1 and Polr2a transcripts in the retinas of Nfe2l1^{OE}, Nfe2l1$^{Retina\ KO}$, and wild-type mice as detected with RNA in situ hybridization (RNA ISH).

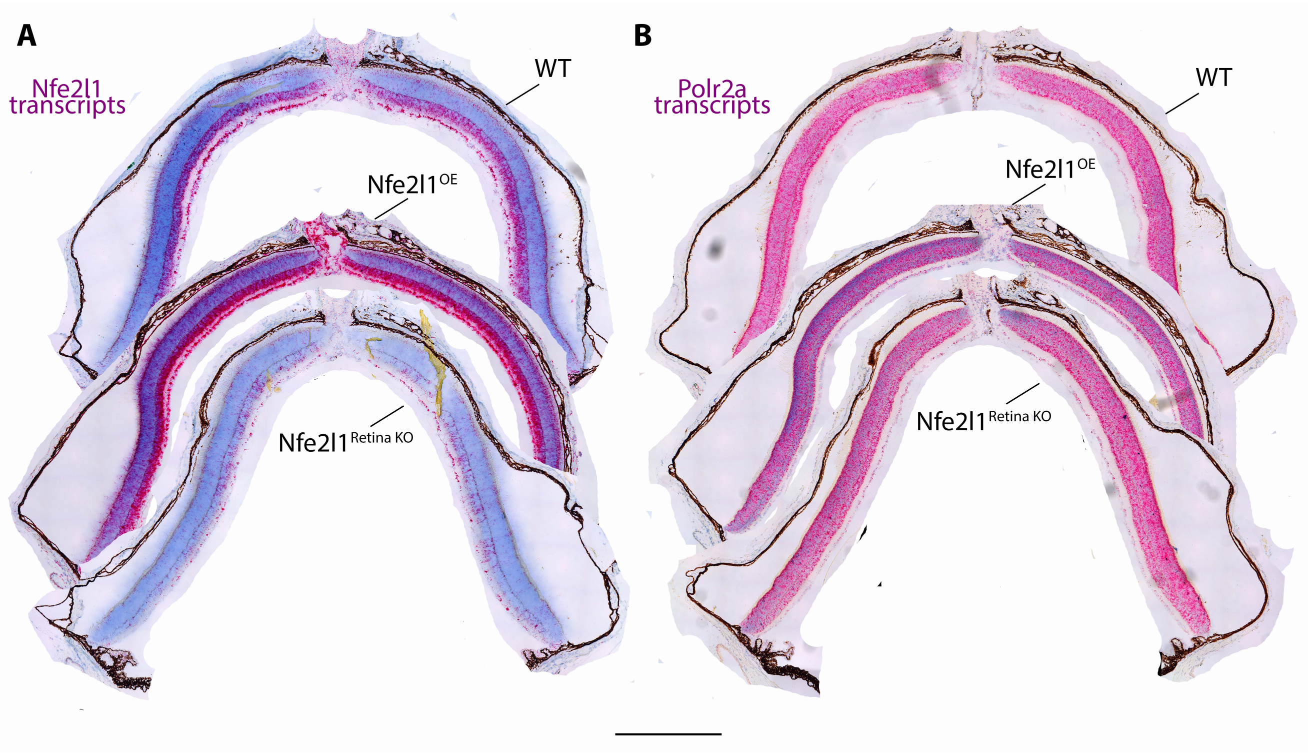

The signals for **(A)** Nfe2l1 or **(B)** Polr2a transcripts appear as red puncta. Samples were processed together using the same conditions. Representative regions of the cross sections from panel **(A)** are shown in Fig. 2A. All mice were one month old. The scale bar is 500 μm.

Fig. S2. Characterization of proteostasis in Nfe2l1 overexpressing mice.

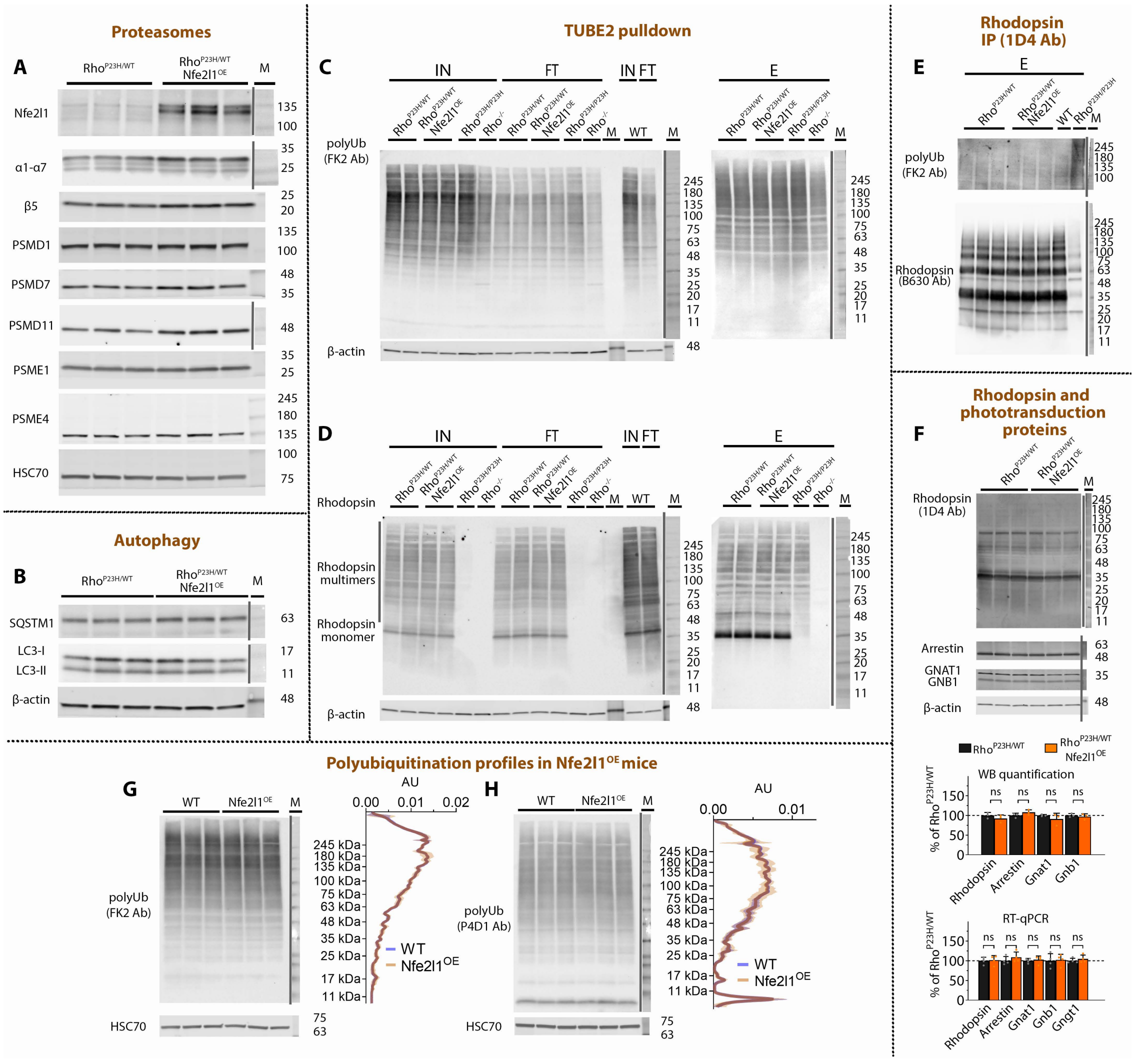

(A, B) Western blots showing the proteasome components and autophagy markers quantified to generate plots in Fig. 3FG. **(C, D)** Analysis of **(C)** ubiquitination and **(D)** rhodopsin in fractions enriched for polyubiquitin with TUBE2 beads from retina lysates of indicated mice as described in Materials in Methods. IN: input; FT: flowthrough; E: eluate. **(E)** Western blot analysis of ubiquitination and rhodopsin in fractions enriched for rhodopsin by immunoprecipitation with 1D4 antibody as descried in Materials in Methods. E: eluate. **(F)** Western blots, quantification plots and RT-qPCR analysis of rhodopsin and major phototransduction proteins (arrestin and transducin subunits) in whole retinas of $Rho^{P23H/WT}/Nfe2l1^{OE}$ and $Rho^{P23H/WT}$ littermate mice. **(G, H)** Western blotting for polyubiquitin chains and density plots as detected with FK2 and P4D1 antibodies in retinal lysates of $Nfe2l1^{OE}$ and WT littermate mice. Color-stained protein markers (M) were typically detected as non-specific bands together with proteins of interest during ECL and IR imaging systems or (if the signal was weak) were added from photographed blots (separated with a gray line). Rhodopsin knockout mice were ten weeks old, $Rho^{P23H/P23H}$ homozygote mice were 14 days old, and other mice were 28 days old.

Fig. S3. Nfe2l1 overexpression improves photoreceptor survival in Rho$^{P23H/WT}$ mice.

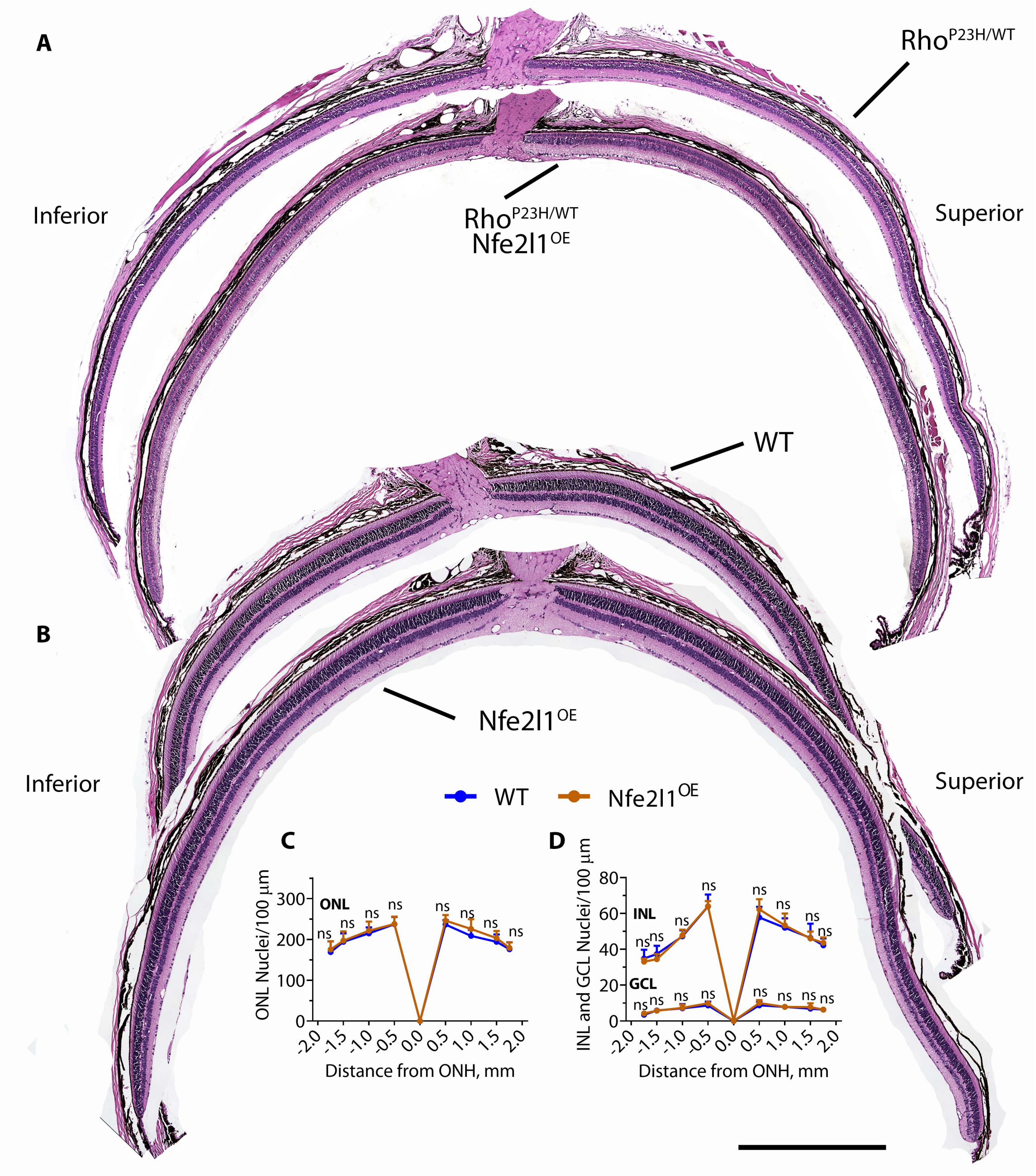

(A, B) The representative images of H&E-stained retinal sections from **(A)** 6-month-old Rho$^{P23H/WT}$/Nfe2l1^{OE} and Rho$^{P23H/WT}$ and **(B)** 12-month-old Nfe2l1^{OE} and WT littermate mice. **(C, D)** Spider diagrams represent the number of nuclei counted in 100-µm segments along the inferior-superior axis in **(C)** ONL or **(D)** INL and GCL at the indicated distances from the center of the optic nerve head (ONH) in 12-month-old Nfe2l1^{OE} (n=10) and WT (n=9) littermate mice. The data are presented as the mean ± SD. The scale bar is 500 µm. ONL: outer nuclear layer (containing photoreceptor nuclei); INL: inner nuclear layer; and GCL: ganglion cell layer. Quantification was performed by individuals not aware of genotype.

Fig. S4. Detection of Nfe2l1 and Polr2a transcripts in the retinas of G$\gamma_1^{-/-}$/Tsc2$^{Rod\ KO}$ and G$\gamma_1^{-/-}$ mice using RNA in situ hybridization (RNA ISH).

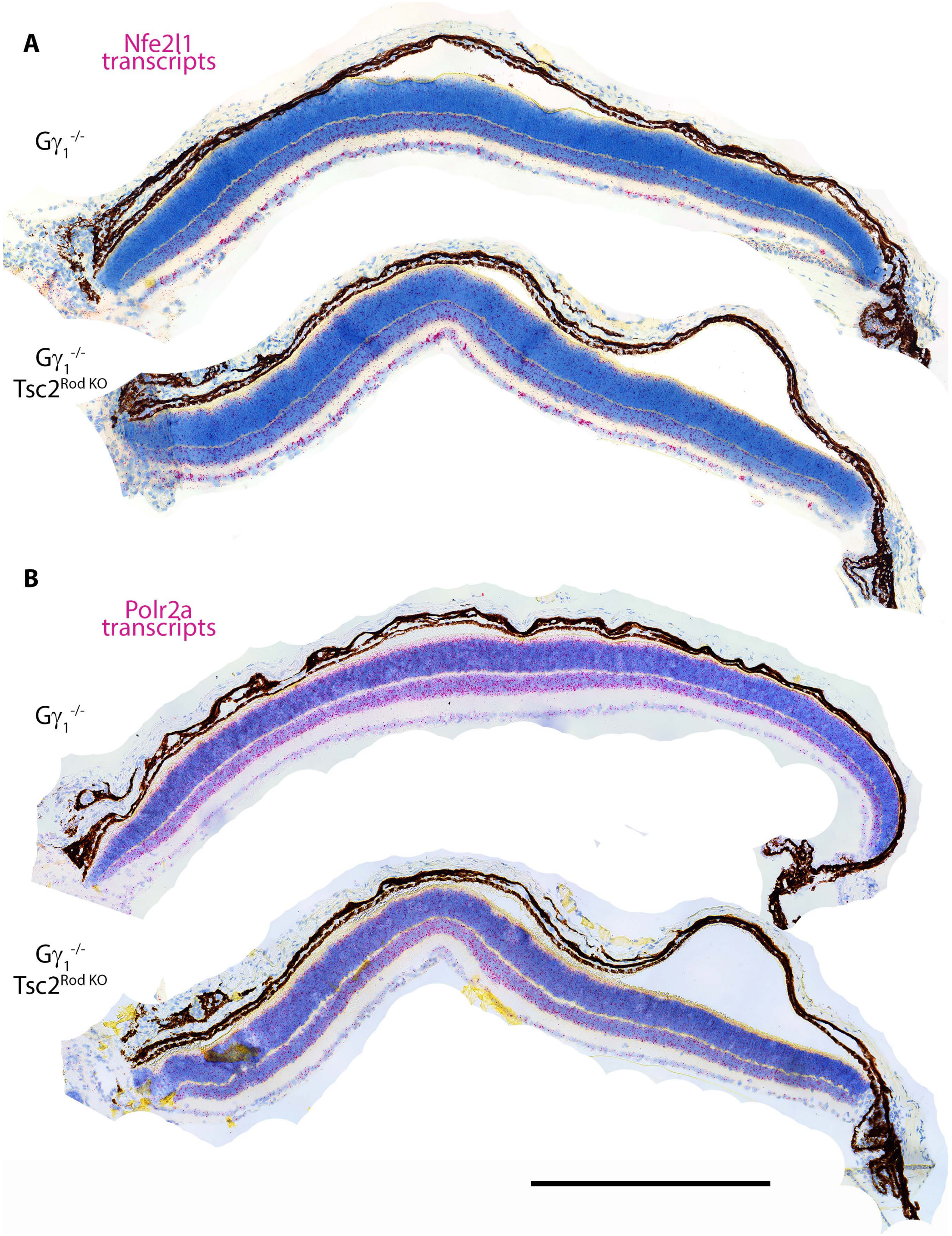

The signals for **(A)** Nfe2l1 or **(B)** Polr2a transcripts appear as red puncta. Samples were processed together using the same conditions. Representative regions of the cross sections stained for Nfe2l1 transcripts are shown in Fig. 7J. The scale bar is 500 μm. All mice were one month old.

Table S1. Statistical analysis for Nfe2l1 and proteasome transcripts in rod scRNAseq dataset. p-Values were calculated using FindMarkers() function with t-test method in Seurat R package. Avg_logFC - log fold-change of the average expression between the two groups; Pct.1 - the percentage of cells where the gene is detected in the first group; Pct.2-the percentage of cells where the gene is detected in the second group; P_val_adj- adjusted p-value calculated based on Bonferroni correction using all genes in the dataset.

	**P_value**	**Avg_log2FC**	**Pct.1**	**Pct.2**	**P_val_adj**
Nfe2l1	0.0048462	0.134	0.101	0.08	1
Psma1	0.0000036	0.208	0.186	0.14	0.068797
Psma2	0.0851614	0.100	0.149	0.134	1
Psma3	0.0003623	0.129	0.336	0.288	1
Psma4	0.0029446	0.145	0.115	0.091	1
Psma5	0.0128809	0.100	0.104	0.085	1
Psma6	0.0000172	0.199	0.091	0.061	0.324335
Psma7	0.0013345	0.102	0.171	0.139	1
Psmb1	0.0001947	0.133	0.153	0.119	1
Psmb2	0.0626371	0.113	0.126	0.111	1
Psmb3	0.0078708	0.115	0.133	0.11	1
Psmb4	0.0004747	0.157	0.148	0.117	1
Psmb5	0.0043499	0.108	0.143	0.118	1
Psmb6	0.0084231	0.114	0.156	0.132	1
Psmb7	0.1070245	0.089	0.098	0.086	1
Psmb8	0.3207471	0.007	0.001	0	1
Psmb9	0.1587898	0.007	0.001	0	1
Psmc1	0.9213380	-0.017	0.082	0.082	1
Psmc2	0.0627628	0.130	0.132	0.117	1
Psmc3	0.7429288	-0.037	0.134	0.131	1
Psmc4	0.1221477	0.025	0.102	0.089	1
Psmc5	0.3212740	-0.011	0.139	0.129	1
Psmc6	0.0440298	0.110	0.324	0.298	1
Psmd1	0.0469124	0.133	0.094	0.08	1
Psmd2	0.3196853	0.065	0.093	0.086	1
Psmd3	0.8344439	-0.004	0.062	0.06	1
Psmd4	0.0334768	0.086	0.185	0.163	1
Psmd5	0.7385508	0.013	0.045	0.043	1
Psmd6	0.0001743	0.185	0.091	0.064	1
Psmd7	0.0000032	0.178	0.163	0.119	0.060456
Psmd11	0.0006223	0.119	0.163	0.13	1
Psmd12	0.0033843	0.106	0.127	0.102	1
Psmd13	0.9543703	-0.017	0.071	0.071	1
Psmd14	0.0123834	0.144	0.077	0.061	1
Psme1	0.5637596	-0.015	0.005	0.006	1
Psme2	0.4539073	0.026	0.02	0.017	1
Psme4	0.2877288	-0.085	0.064	0.071	1

Table S2. Breeding strategies and littermates used in experiments.

Line abbreviation	Breeding strategy to establish line	Littermates used in experiments
WT (C57BL/6J)	Jackson Lab (Stock# 000664)	
$Nfe2l1^{OE}$	Nfe2l1 overexpressing transgene × WT	$Nfe2l1^{OE}$ vs WT littermates
$Nfe2l1^{Retina\ KO}$	$Nfe2l1^{fl/fl}$ × Chx10-Cre	$Nfe2l1^{fl/fl}$ vs $Nfe2l1^{fl/fl}$/Chx10-Cre(+)
$Rho^{P23H/WT}/Nfe2l1^{OE}$	$Rho^{P23H/P23H}$ × $Nfe2l1^{OE}$	$Rho^{P23H/WT}/Nfe2l1^{OE}$ vs $Rho^{P23H/WT}$
$Nfe2l1^{OE}/Ub^{G76V}$- GFP	$Nfe2l1^{OE}$ × Ub^{G76V}- GFP	Line was used for breeding with $Rho^{P23H/P23H}$ mice
$Rho^{P23H/WT}/Nfe2l1^{OE}/Ub^{G76V}$-GFP	$Rho^{P23H/P23H}$ × $Nfe2l1^{OE}/Ub^{G76V}$- GFP	$Rho^{P23H/WT}/Nfe2l1^{OE}/Ub^{G76V}$-GFP(+/-) vs $Rho^{P23H/WT}/Ub^{G76V}$- GFP(+/-)
$G\gamma_1^{-/-}/Tsc2^{Rod\ KO}$	$G\gamma_1^{-/-}$× $Tsc2^{fl/fl}$ × iCre75	$G\gamma_1^{-/-}/Tsc2^{fl/fl}$ vs $G\gamma_1^{-/-}/Tsc2^{fl/fl}$/iCre75
$G\gamma_1^{-/-}/Tsc2^{Rod\ KO}/Ub^{G76V}$-GFP	$G\gamma_1^{-/-}$×$Tsc2^{fl/fl}$ × iCre75×Ub^{G76V}- GFP	$G\gamma_1^{-/-}/Tsc2^{fl/fl}/Ub^{G76V}$- GFP(+/-) vs $G\gamma_1^{-/-}/Tsc2^{fl/fl}$/iCre75/$Ub^{G76V}$-GFP(+/-)
$G\gamma_1^{-/-}/Nfe2l1^{OE}$	$G\gamma_1^{-/-}$× $Tsc2^{fl/fl}$ × $Nfe2l1^{OE}$	$G\gamma_1^{-/-}/Tsc2^{fl/fl}$ vs $G\gamma_1^{-/-}/Tsc2^{fl/fl}/Nfe2l1^{OE}$
$G\gamma_1^{-/-}/Nfe2l1^{OE}/Ub^{G76V}$-GFP	$G\gamma_1^{-/-}$×$Tsc2^{fl/fl}$ × $Nfe2l1^{OE}$ ×Ub^{G76V}- GFP	$G\gamma_1^{-/-}/Tsc2^{fl/fl}/Ub^{G76V}$- GFP(+/-) vs $G\gamma_1^{-/-}/Tsc2^{fl/fl}$/ $Nfe2l1^{OE}/Ub^{G76V}$-GFP(+/-)

Table S3. Antibodies and probes used for western blotting and immunolocalization studies.

Antibody	Manufacturer or source, Catalog Number	Host Species	Dilution	Blocking	Detection Method	Detection Reagent
LAMIN A/C	Novus, NB100-56649SS	Rabbit	1:5000	TBST (20 mM Tris-HCl, pH 8.0, 0.1% Tween-20) containing 5% Cytiva RPN418	ECL	Bio-Rad #1705061
LC3 A/B	Cell Signaling, 4108	Rabbit	1:5000			
p62 (SQSTM1)	Novus, NBP1-48320SS	Rabbit	1:5000			
Proteasome 20S α1, 2, 3, 5, 6 & 7 subunits	Enzo Life Sciences, BML-PW8195-0100	Mouse	1:5000			
PSMD11	Novus, NBP1-46192	Rabbit	1:5000			
POLYUBIQUITIN (FK2)	Millipore, ST1200-100UG	Mouse	1:5000			
UBIQUITIN (P4D1)	UBPBio, Y3011	Mouse	1:5000			
RHODOPSIN	Santa Cruz, sc-57432	Mouse	1:1000			
TCF11/NRF1	Cell Signaling, 8052S	Rabbit	1:5000	Bio-Rad 12010020		Bio-Rad #1705062
Peroxidase-conjugated AffiniPure Donkey Anti Rabbit IgG (H+L)	Jackson Immuno Research, 715-035-152	Donkey	1:20000	NA		NA
Peroxidase-conjugated AffiniPure Donkey Anti Mouse IgG (H+L)	Jackson Immuno Research, 715-035-150	Donkey	1:20000	NA		NA
Alexa Flour 790-Conjugated AffiniPure Donkey Anti-Mouse IgG (H+L)	Jackson Immuno Research, 715-655-150	Donkey	1:10000	NA	Li-Cor near-Infrared	NA
Alexa Fluor 680-conjugated AffiniPure Donkey AntiRabbit IgG (H+L)	Jackson Immuno Research, 711-625-152	Donkey	1:10000	NA		NA
ARRESTIN	Dr. Clay Smith, C10C10	Mouse	1:250	Li-Cor 927-60001		NA
beta Actin Loading Control Monoclonal Antibody, DyLight 800 4X PEG	Invitrogen, BA3R, MA5-15739-D800	NA	1:10000			NA
HSC70	ENZO, ADI-SPA-819-F	Rabbit	1:10000			NA
GFP	Novus, NB600-308	Rabbit	1:5000			NA
GNAT1 (Xenopus)	Dr. Clay Smith	Rabbit	1:250			NA
GNB1 (Bovine)	Dr. Vadim Arshavsky	Sheep	1:250			NA
PSMB5	Abcam, ab3330	Rabbit	1:5000			NA
PSMD1	Abcam, ab140682	Rabbit	1:5000			NA
PSMD7	Abcam, ab140428	Rabbit	1:5000			NA
PSME1	Enzo Life Sciences, BML-PW8185-0100	Rabbit	1:5000			NA
PSME4	Invitrogen, PA1-1961	Rabbit	1:5000			NA
RHODOPSIN	Santa Cruz, sc-57432	Mouse	1:1000			NA
RHODOPSIN	Dr. Clay Smith, B630	Mouse	1:250			NA
VIMENTIN	Abcam, ab92547	Rabbit	1:5000			NA
WGA (wheat germ agglutinin) Alexa Fluor 555 conjugate	Thermo Fisher, W32464	NA	1:500	NA	Confocal Microscopy	NA

Table S4. RT-qPCR primer sets.

Gene	Forward Primer	Reverse Primer
Actb	cgagcacagcttctttgcag	ttcccaccatcacaccctgg
Gnat1	tcatcgctacttcgccacaa	atgtagttgccggcatcctc
Gnb1	gcctgtctcttgctcctgac	gtcaaacagcctgcatgtgg
Gngt1	aggaagtgacactggagaga	aatcacacagcctcctttga
Hsc70	caagcgcaataccaccatcc	tgcctgtgagctcgaacttt
Nfe2l1	gagttccttcggtctctgcg	catcgtgcgaggaatgagga
Psma1	ccagatcccaacacagcgat	gagctgattgagaacgggct
Psma5	aggtgctatgtctcgtccct	ccctcagaagcagagccaat
Psmb5	ccatgatctgtggctgggat	cctcgatccataacgccgta
Psmc4	ccagataagatttcaggagccga	tcttgatcacggtcttgtatgct
Psmc6	acactggatcctgctttgct	tcaccatgctttgtaataggtcc
Psmd1	accatccacattcgcatatcct	cacttccatcttctcctcctcc
Psmd11	atgacccaatcatcagcacac	ctactgggggttcatcgaaaatg
Psme1	gtcactacctggttgcagct	acttggagatctgcgtgtgg
Psme4	ccccagctccttatgaacctc	ctgctgcttgtgttcttgcc
Rho	atctcgagggcttctttgcc	cggaagttgctcatcggctt
Sag	gagaaagaagaggcatcgcg	ctcctagaaagcccgacacc

Table S5. RNA *in situ* hybridization (RNA ISH) probes used to visualize RNA molecules and as controls on paraffin-embedded retinal sections.

**Gene**	**Reference number**	**LOT number**	**Manufacturer**
Mm-Nfe2l1	580618	22234B	Advanced Cell Diagnostics, Inc
Mm-Polr2a (positive control)	312478	22237B	
dapB (negative control)	312038	22236A	

Data S1. (separate Excel Microsoft file)

DEGs (differentially expressed genes) in retinas of Nfe2l1 overexpressing mice in comparison to WT littermates identified via bulk RNAseq. Data were normalized using DESeq2. p-Values represent false discovery rate. Genes with p-Values <0.05 were considered differentially expressed. Gene annotation was performed using STRING (string-db.org). Log10(FC) column represents the fold changes (FC) in the expression levels of the indicated gene in $Nfe2l1^{OE}$ mice in comparison to WT littermates. "Retina expression" column represents the major retinal cell type(s) containing the transcripts of the indicated gene, which was assessed using in-house single-cell retina datasets. " Nfe2l1 Target?" column indicates genes potentially regulated/targeted by Nfe2l1 identified based on literature.

Data S2. (separate Excel Microsoft file)

DEGs (differentially expressed genes) in livers of Nfe2l1 overexpressing mice in comparison to WT littermates identified via bulk RNAseq (Fig. 2EFG) and results of GO or IPA pathway. Data were normalized using DESeq2. p-Values represent false discovery rate. Genes with p-Values <0.05 were considered differentially expressed. Gene annotation was performed using STRING (string-db.org). Log10(FC) represents the fold changes in the expression levels of indicated genes in $Nfe2l1^{OE}$ mice in comparison to WT littermates.
